# Computing degree based topological indices of algebraic hypergraphs

**DOI:** 10.1016/j.heliyon.2024.e34696

**Published:** 2024-07-22

**Authors:** Amal S. Alali, Esra Öztürk Sözen, Cihat Abdioğlu, Shakir Ali, Elif Eryaşar

**Affiliations:** a Department of Mathematical Sciences, College of Science, Princess Nourah bint Abdulrahman University, P. O. Box-84428, Riyadh-11671, Saudi Arabia; b Department of Mathematics, Sinop University, 57000, Sinop, Turkey; c Department of Mathematics and Science Education, Karamanoğlu Mehmetbey University, Karaman, Turkey; d Department of Mathematics, Faculty of Science, Aligarh Muslim University, Aligarh, India

**Keywords:** 13A70, 05C07, 05C09, 05C25, 05C65, Commutative ring, Hypergraph, Prime ideal sum hypergraph(PISH), Vertex degree, Topological indices

## Abstract

Topological indices are numerical parameters that indicate the topology of graphs or hypergraphs. A hypergraph H=(V(H),E(H)) consists of a vertex set V(H) and an edge set E(H), where each edge e∈E(H) is a subset of V(H) with at least two elements. In this paper, our main aim is to introduce a general hypergraph structure for the prime ideal sum (PIS)- graph of a commutative ring. The prime ideal sum hypergraph of a ring *R* is a hypergraph whose vertices are all non-trivial ideals of *R* and a subset of vertices $E_{i}$ with at least two elements is a hyperedge whenever I+J is a prime ideal of *R* for each non-trivial ideal *I*, *J* in $E_{i}$ and $E_{i}$ is maximal with respect to this property. Moreover, we also compute some degree-based topological indices (first and second Zagreb indices, forgotten topological index, harmonic index, Randić index, Sombor index) for these hypergraphs. In particular, we describe some degree-based topological indices for the newly defined algebraic hypergraph based on prime ideal sum for $Z_{n}$ where $n=p^{\alpha },pq,p^{2}q,p^{2}q^{2},pqr,p^{3}q$, $p^{2}qr,pqrs$ for the distinct primes p,q,r and *s*.

## 1 Introduction

Graph theory is related to the modeling of the objects and relations of each object with each other by representing each object as a vertex and each relation of objects as an edge. Hyperstructure theory came into sight in 1934 as a general version of structures. While working as element-based level in algebraic systems, the opportunity of working on sets is got in hypersystems owing to this theory [1]. With this difference, hyperstructures have more strong applications on complex hypernetworks first, and also on many applied sciences including informatics, system modeling, social network analysis, system engineering, web information systems, architecture and etc. In particular, in [2], the authors interested in investigation of general actions and applications on hyperstructures as a generalization of the classical concept of a discrete dynamical system. The theoretic frame of hyperstructures in automata, cryptography and artificial intelligence is given in [3]. In [4], the authors emphasized the importance of hypergraphs in optimization theory. Hypergraph theory was introduced by French mathematician Berge as a generalization of graph theory in [5]. A graph G=(V(G),E(G)) is a pair of sets, where V(G) indicates the set of finitely many vertices and E(G) indicates the set of edges connecting related vertices [6]. A hypergraph H=(V(H),E(H)) is a pair of sets where V(H) indicates the set of finitely many vertices and E(H) indicates the finitely many sets of edges $E_{i}(1\leq i\leq n)$, provided $\cup_{i=1}^{n}E_{i}=V(H)$, whose elements consists of non-empty subsets of V(H). While an edge can link two related nodes each other in a graph, a hyperedge can include vertices more than two in a hypergraph. From this aspect, hypergraphs are more stronger than graphs for describing complex systems. A vertex of a hypergraph is said to be isolated if there exists no hyperedge containing it, and a hypergraph containing no hyperedge covering any of the vertices is called null. In a hypergraph, two different vertices contained by the same hyperedge are called adjacent. If there is no hyperedge contained by the others, then the hypergraph is called simple. A hypergraph is called linear if every two distinct edges of *H* intersect in at most one vertex.

Defining zero divisor graphs over commutative rings was the beginning of graph theory in pure algebra. In [7], the authors investigated some graph theoretic parameters such as girth and diameter for the zero divisor graph of a commutative ring. Also in [8] these parameters were investigated for a graph of commutative ring with non-zero divisors which satisfy comparability conditions between ideals or prime ideals of the ring. In [9], the coloring problem was handled for zero divisor graph of a commutative ring. Afterward, handling different graph-theoretic properties of algebraic structures has been an attractive field for many researchers. It is possible to collect some graphical structures of rings in the literature as follows: Cayley graphs [10], total [11] and regular graphs [12], annihilating ideal graphs [13], (generalized [14]) comaximal graphs [15] are the leading ones of the studies taking place in this category. In addition to these, as ideal theory is the heart of the ring theory, also some ideal-based graphs have been defined on commutative rings. For example, inclusion ideal graph [16], intersection graphs of ideals [17], co-maximal ideal graphs [18], prime ideal sum (PIS)- graphs [19] are some of them. In this article, we define a new algebraic hypergraph modeling over commutative rings with unity.

Topological indices are the general name for a mathematical operation that converts a graph structure to a number and actually characterized the topology of a graph. There are many types of topological indices, and topological indices based on point degree are widely used today. In [20], Banhatti type degree based topological indices were created. Certain degree based topological indices were used for QSPR analysis of some alkane derivatives [21], [22], [23] and also used for investigating the topological structure of another chemical compounds such as [24], [25], [26]. In [27] inequalities between vertex-degree-based topological indices were studied. Many vertex-degree-based topological indices have been introduced so far [28]. Among the oldest and most studied degree-based topological indices are the first Zagreb index and the second Zagreb index. Zagreb indices were introduced in [29] by Gutman et al. Followed by the first and second Zagreb indices, Furtula and Gutman [30] introduced and studied forgotten topological index. The harmonic index is another degree-based topological index [31]. Favaron et al. [32] considered the relation between the harmonic index and the eigenvalues of graphs. The Randić index, proposed by Randić [33] and this index is one of the most successful vertex degree-based molecular descriptors (topological indices) in the structure property and structure activity relationship studies [34]. In 2020, Gutman introduced a new vertex-degree-based topological index defined which was named as Sombor index. Some basic properties of the as Sombor index were established. Geometric meaning of this index and the relations between Sombor and the other degree based topological indices were studied in [35] and [36], respectively.

Recently, while interest in graph modeling of algebraic structures has increased, the determination of graph parameters has also become one of the researched problems. Topological index calculations, specifically graph-parametric index, shed light on the topological properties of algebraic structures. In 2022, Hamzekolaee and Norouzi studied the hypergraph characterizations and parameters of a number of finite modules and showed that hypergraph modeling of algebraic graphs is also possible [37]. In 2023, Selvakumar et al. defined the 3-zero-divisor hypergraph of commutative rings and studied their topological properties [38]. So far, there has not been much work on the degree-based topological indices of hypergraphs.

Based on these studies in the literature, a hypergraph interpretation of prime ideal sum graphs, previously defined by [19] and then topologically analyzed by [39], was made. The prime ideal sum hypergraph of a ring *R* is a hypergraph whose vertices are all non-trivial ideals of *R* and a subset of vertices $E_{i}$ with at least two elements is a hyperedge whenever I+J is a prime ideal of *R* for each non-trivial ideal *I*, *J* in $E_{i}$ and $E_{i}$ is maximal with respect to this property. Moreover, we compute some degree-based topological indices (first and second Zagreb indices, forgotten topological index, harmonic index, Randić index, sombor index) for these hypergraphs. Preciely, we describe some degree-based topological indices for the newly defined algebraic hypergraph based on prime ideal sum for $Z_{n}$ where $n=p^{\alpha },pq,p^{2}q,p^{2}q^{2},pqr,p^{3}q$, $p^{2}qr,pqrs$ for the distinct primes p,q,r and *s*.

## 2 Preliminaries

In this section, we give a list of definitions which we need in the subsequent sections. We begin with the following definition.

Definition 2.1 Let H=(V(H),E(H)) be a hypergraph. For each v∈V, the degree of *v*, denoted by $d_{v}$, is the number of edges that contain it [40].

Definition 2.2 The prime ideal sum graph (abbreviated by *PIS*) of a ring is described as a graph whose vertices are all non-trivial ideals of the ring and edges connect two vertices whenever the sum of these vertices is a prime ideal.

Definition 2.3 For a ring *R*, the prime ideal sum hypergraph of *R*, abbreviated by $PISH(R)=(V_{H}(R),E_{H}(R))$, is a hypergraph whose vertices are all non-trivial ideals of *R* and a subset of vertices $E_{i}\in E_{H}(R)$ with at least two elements is a hyperedge of PISH(R) provided I+J is a prime ideal of *R* for each non-trivial ideal *I*, *J* in $E_{i}$ and $E_{i}$ is maximal with respect to this property.

**Figure 1:**
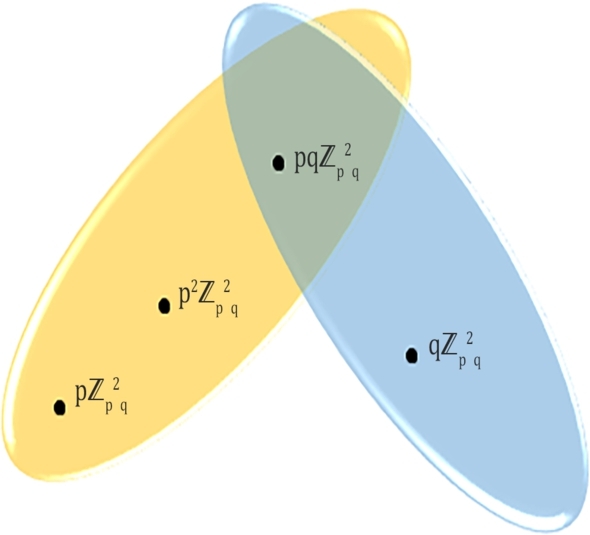
*PISH* structure of $Z_{p^{2}q}$.

**Figure 2:**
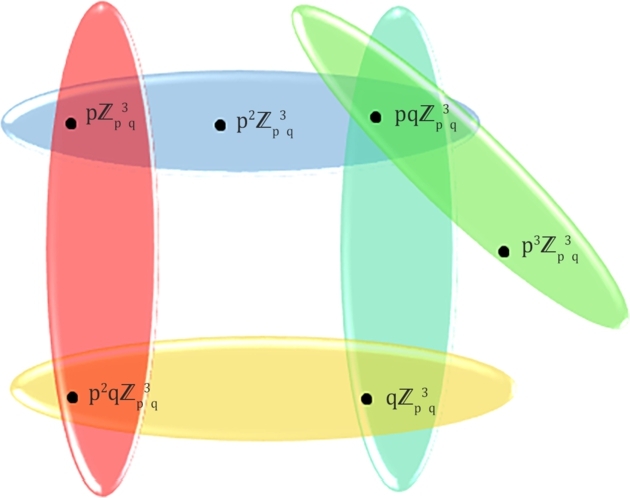
*PISH* structure of $Z_{p^{3}q}$.

**Figure 3:**
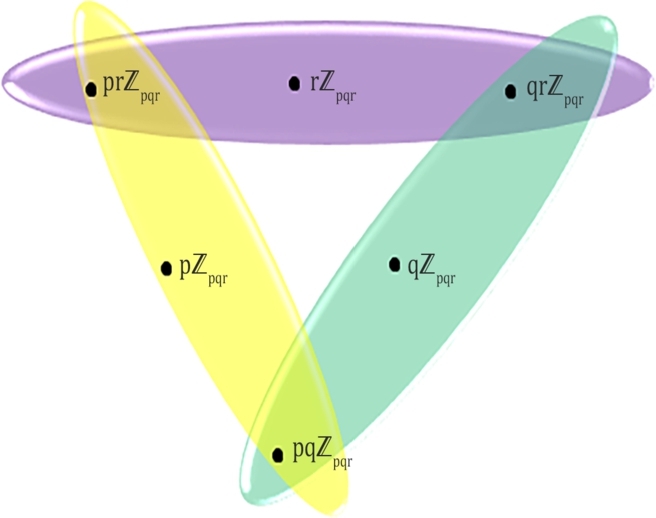
*PISH* structure of $Z_{pqr}$.

For example, see Fig. 1, Fig. 2 and Fig. 3 for the PIS-hypergraph (abbreviated by PISH) representations for $n=p^{2}q,p^{3}q,pqr$ in $Z_{n}$, respectively.

With these notions, we give our modified definitions for some degree-based topological indices of H=(V(H),E(H)) hypergraphs as follows:

$$\text{First Zagreb Index:}\;M_{1}(H)=\sum_{v\in V(H)}{\left(d_{u}\right)}^{2},$$

$$\text{Second Zagreb Index:}\;M_{2}(H)=\sum_{uv\in E(H)}d_{u}d_{v},$$

$$\text{Forgotten Topological Index:}\;F(H)=\sum_{v\in V(H)}{\left(d_{u}\right)}^{3},$$

$$\text{Harmonic Index:}\;H(H)=\sum_{uv\in E(H)}\frac{2}{d_{u}+d_{v}},$$

$$\text{Randić Index:}\;R(H)=\sum_{uv\in E(H)}\frac{1}{\sqrt{d_{u}d_{v}}},$$

$$\text{Sombor Index:}\;SO(H)=\sum_{uv\in E(H)}\sqrt{{\left(d_{u}\right)}^{2}+{\left(d_{v}\right)}^{2}}.$$

## 3 The results

### 3.1 Degree-based topological indices of PIS-hypergraphs

The first main result of this paper is the following theorem.

Theorem 1 *Let p a prime number and*H=(V(H),E(H))*be the PIS-hypergraph of*$Z_{p^{\alpha }}$*for*α≥2*. Then,*

$$\begin{array}{ccc} M_{1}(H)=\alpha^{2}-\alpha -1,\; & M_{2}(H)=(\alpha -2)(\alpha -1),\; & F(H)=\alpha^{3}-3\alpha^{2}+4\alpha -3\\ H(H)=\frac{2\alpha -4}{\alpha },\; & R(H)=\frac{\alpha -2}{\sqrt{\alpha -1}},\; & SO(H)=(\alpha -2)\sqrt{\alpha^{2}-2\alpha +2} \end{array}.$$

Proof Let us list all ideals of $Z_{p^{\alpha }}$. Then we have {0}, $p^{\alpha -1}Z_{p^{\alpha }}$, $p^{\alpha -2}Z_{p^{\alpha }}$, ... $,p^{3}Z_{p^{\alpha }}$, $p^{2}$ $Z_{p^{\alpha }}$, $pZ_{p^{\alpha }}$, $Z_{p^{\alpha }}$ and we take $u_{1}=pZ_{p^{\alpha }}$, $u_{2}=p^{2}pZ_{p^{\alpha }}$, ..., $u_{\alpha -1}=p^{\alpha -1}pZ_{p^{\alpha }}$. Note that $u_{1}$ is the unique prime ideal because of the locality of the ring. Thus, *PIS*-hypergraph H=(V(H),E(H)) consists of set of the vertices $V(H)=\{u_{1}$, $u_{2}$, $u_{3}$, ..., $u_{\alpha -1}\}$ and set of the edges $E(H)=\{E_{1}=\{u_{1},u_{2}\}$, $E_{2}=\{u_{1},u_{3}\},...$, $E_{\alpha -1}=\{u_{1},u_{\alpha -1}\}\}$. Therefore, $d(u_{1})=\alpha -1$, $d(u_{2})=1,d(u_{3})=1,...,d(u_{\alpha -1})=1$ are obtained by vertex partition technique. Then, we obtain

$$M_{1}(H)=\sum_{v\in V(H)}{\left(d_{u}\right)}^{2}={\left(\alpha -1\right)}^{2}+\underset{\alpha -2\text{ times}}{\underset{︸}{1^{2}+...+1^{2}}}={\left(\alpha -1\right)}^{2}+\left(\alpha -2\right)=\alpha^{2}-\alpha -1,$$

$$M_{2}(H)=\sum_{uv\in E(H)}d_{u}d_{v}=\underset{\alpha -2\text{ times}}{\underset{︸}{(\alpha -1)1+(\alpha -1)1+...+(\alpha -1)1}}=(\alpha -2)(\alpha -1),$$

$$F(H)=\sum_{v\in V(H)}{\left(d_{u}\right)}^{3}={\left(\alpha -1\right)}^{3}+\underset{\alpha -2\text{ times}}{\underset{︸}{1^{3}+...+1^{3}}}={\left(\alpha -1\right)}^{3}+\left(\alpha -2\right)=\alpha^{3}-3\alpha^{2}+4\alpha -3,$$

$$H(H)=\sum_{uv\in E(H)}\frac{2}{d_{u}+d_{v}}=\underset{\alpha -2\text{ times}}{\underset{︸}{\frac{2}{\left(\alpha -1\right)+1}+\frac{2}{\left(\alpha -1\right)+1}+...+\frac{2}{\left(\alpha -1\right)+1}}}=\frac{2\alpha -4}{\alpha },$$

$$R(H)=\sum_{uv\in E(H)}\frac{1}{\sqrt{d_{u}d_{v}}}=\underset{\alpha -2\text{ times}}{\underset{︸}{\frac{1}{\sqrt{\left(\alpha -1\right)1}}+\frac{1}{\sqrt{\left(\alpha -1\right)1}}+...+\frac{1}{\sqrt{\left(\alpha -1\right)1}}}}=\frac{\alpha -2}{\sqrt{\alpha -1}},$$

$$SO(H)=\sum_{uv\in E(H)}\sqrt{{\left(d_{u}\right)}^{2}+{\left(d_{v}\right)}^{2}}=\underset{\alpha -2\text{ times}}{\underset{︸}{\sqrt{1^{2}+{\left(\alpha -1\right)}^{2}}+\sqrt{1^{2}+{\left(\alpha -1\right)}^{2}}+...+\sqrt{1^{2}+{\left(\alpha -1\right)}^{2}}}}=\left(\alpha -2\right)\sqrt{\alpha^{2}-2\alpha +2}.$$

 □

Theorem 2 *Let p, q be distinct prime numbers and*H=(V(H),E(H))*be the PIS-hypergraph of*$Z_{pq}$*. The values of all the degree-based topological indices mentioned above for*$H(Z_{pq})$*are* 0*.*

Proof Since $H(Z_{pq})$ is a null hypergraph, all points have a degree of 0 and therefore their index values are also 0. □

Theorem 3 *Let p, q be distinct prime numbers and* H=(V(H),E(H)) *be the PIS-hypergraph of* $Z_{p^{2}q}$ *. Then,*

$$\begin{array}{ccc} M_{1}(H)=7\; & M_{2}(H)=7\; & F(H)=11\\ H(H)=3\; & R(H)=3.12\; & SO(H)=8.12 \end{array}.$$

Proof Let us consider the ideals $u_{1}=pZ_{p^{2}q}$, $u_{2}=qZ_{p^{2}q}$, $u_{3}=pqZ_{p^{2}q}$, $u_{4}=p^{2}Z_{p^{2}q}$, where $u_{1}$, $u_{2}$ are prime ones. Thus *PIS*-hypergraph H=(V(H),E(H)) consists of the sets of vertices and hyperedges such that $V(H)=\{u_{1}$, $u_{2}$, $u_{3}$, $u_{4}\}$ and $E(H)=\{E_{1}=\{u_{1},u_{3},u_{4}\}$, $E_{2}=\{u_{2},u_{3}\}\}$. Since $d(u_{1})=1$, $d(u_{2})=1,d(u_{3})=2,d(u_{4})=1$ are obtained by vertex partition technique, we have

$$M_{1}(H)=\sum_{v\in V(H)}{\left(d_{u}\right)}^{2}=3.1^{2}+2^{2}=7,$$

$$M_{2}(H)=\sum_{uv\in E(H)}d_{u}d_{v}=1.1+2.1+1.2+1.2=7,$$

$$F(H)=\sum_{v\in V(H)}{\left(d_{u}\right)}^{3}=3.1^{3}+2^{3}=11,$$

$$H(H)=\sum_{uv\in E(H)}\frac{2}{d_{u}+d_{v}}=\frac{2}{1+1}+\frac{2}{2+1}+\frac{2}{1+2}+\frac{2}{1+2}=3,$$

$$R(H)=\sum_{uv\in E(H)}\frac{1}{\sqrt{d_{u}d_{v}}}=\frac{1}{\sqrt{1.1}}+\frac{1}{\sqrt{2.1}}+\frac{1}{\sqrt{1.2}}+\frac{1}{\sqrt{1.2}}=3.12.$$

$$SO(H)=\sum_{uv\in E(H)}\sqrt{{\left(d_{u}\right)}^{2}+{\left(d_{v}\right)}^{2}}=\sqrt{1^{2}+1^{2}}+\sqrt{2^{2}+1^{2}}+\sqrt{1^{2}+2^{2}}+\sqrt{1^{2}+2^{2}}$$

$$=\sqrt{2}+\sqrt{5}+\sqrt{5}+\sqrt{5}=8.12.$$

 □

Theorem 4 *Let p, q, r be distinct prime numbers and* H=(V(H),E(H)) *be the PIS-hypergraph of* $Z_{pqr}$ *. Then,*

$$\begin{array}{ccc} M_{1}(H)=15\; & M_{2}(H)=24\; & F(H)=27\\ H(H)=5.5\; & R(H)=3.12\; & SO(H)=21.90 \end{array}.$$

Proof Let us consider the non-trivial ideals $u_{1}=pZ_{pqr}$, $u_{2}=qZ_{pqr}$, $u_{3}=rZ_{pqr}$, $u_{4}=pqZ_{pqr},u_{5}=prZ_{pqr},u_{6}=qrZ_{pqr}$ where $u_{1}$, $u_{2}$, $u_{3}$ are the only prime ones. Thus, *PIS*-hypergraph H=(V(H),E(H)) consists of $V(H)=\{u_{1}$, $u_{2}$, $u_{3}$, $u_{4},u_{5},u_{6}\}$ and $E(H)=\{E_{1}=\{u_{1},u_{4},u_{5}\}$, $E_{2}=\{u_{2},u_{4},u_{6}\}$, $E_{3}=\{u_{3}$, $u_{5}$, $u_{6}\}\}$ as the sets of vertices and hyperedges, respectively. The values, $d(u_{1})=1$, $d(u_{2})=1,d(u_{3})=1,d(u_{4})=2,d(u_{5})=2,d(u_{6})=2$ are obtained by vertex partition technique. Then,

$$M_{1}(H)=\sum_{v\in V(H)}{\left(d_{u}\right)}^{2}=3.1^{2}+3.2^{2}=15,$$

$$M_{2}(H)=\sum_{uv\in E(H)}d_{u}d_{v}=1.2+1.2+2.2+1.2+1.2+2.2+1.2+1.2+2.2=24,$$

$$F(H)=\sum_{v\in V(H)}{\left(d_{u}\right)}^{3}=3.1^{3}+3.2^{3}=27,$$

$$H(H)=\sum_{uv\in E(H)}\frac{2}{d_{u}+d_{v}}=\frac{2}{1+2}+\frac{2}{1+2}+\frac{2}{2+2}+\frac{2}{1+2}+\frac{2}{1+2}+\frac{2}{2+2}+\frac{2}{1+2}+\frac{2}{1+2}+\frac{2}{2+2}=5.5$$

$$R(H)=\sum_{uv\in E(H)}\frac{1}{\sqrt{d_{u}d_{v}}}=\frac{1}{\sqrt{1.2}}+\frac{1}{\sqrt{1.2}}+\frac{1}{\sqrt{2.2}}+\frac{1}{\sqrt{1.2}}+\frac{1}{\sqrt{1.2}}+\frac{1}{\sqrt{2.2}}+\frac{1}{\sqrt{1.2}}+\frac{1}{\sqrt{1.2}}+\frac{1}{\sqrt{2.2}}=5.74.$$

$$SO(H)=\sum_{uv\in E(H)}\sqrt{{\left(d_{u}\right)}^{2}+{\left(d_{v}\right)}^{2}}=\sqrt{1^{2}+2^{2}}+\sqrt{1^{2}+2^{2}}+\sqrt{2^{2}+2^{2}}+\sqrt{1^{2}+2^{2}}+\sqrt{1^{2}+2^{2}}+$$

$$\sqrt{2^{2}+2^{2}}+\sqrt{1^{2}+2^{2}}+\sqrt{1^{2}+2^{2}}+\sqrt{2^{2}+2^{2}}=21.90.$$

 □

Theorem 5 *Let p, q be distinct prime numbers and* H=(V(H),E(H)) *be the PIS-hypergraph of* $Z_{p^{2}q^{2}}$ *. Then,*

$$\begin{array}{ccc} M_{1}(H)=38\; & M_{2}(H)=76\; & F(H)=94\\ H(H)=5.93\; & R(H)=6.08\; & SO(H)=47.37 \end{array}.$$

Proof Let us consider the ideals $u_{1}=pZ_{p^{2}q^{2}}$, $u_{2}=qZ_{p^{2}q^{2}}$, $u_{3}=pqZ_{p^{2}q^{2}},u_{4}=p^{2}q$$Z_{p^{2}q^{2}},u_{5}=pq^{2}Z_{p^{2}q^{2}},u_{6}=p^{2}Z_{p^{2}q^{2}},u_{7}=q^{2}Z_{p^{2}q^{2}}$ where $u_{1}$, $u_{2}$ are prime ideals. Thus *PIS*-hypergraph H=(V(H),E(H)) consists of the vertex set $V(H)=\{u_{1}$, $u_{2}$, $u_{3}$, $u_{4},u_{5},u_{6},u_{7}\}$ and hyperedge set $E(H)=\{E_{1}=\{u_{1},u_{3},u_{6}\}$, $E_{2}=\{u_{1},u_{5},u_{6}\}$, $E_{3}=\{u_{2}$, $u_{3}$, $u_{7}\},E_{4}=\{u_{2},u_{4},u_{7}\},E_{5}=\{u_{1},u_{4}\},E_{6}=\{u_{2},u_{5}\}\}$. Also, $d(u_{1})=3$, $d(u_{2})=3,d(u_{3})=2,d(u_{4})=2,d(u_{5})=2,d(u_{6})=2,d(u_{7})=2$ are obtained by vertex partition technique. Thus, we have

$$M_{1}(H)=\sum_{v\in V(H)}{\left(d_{u}\right)}^{2}=2.3^{2}+5.2^{2}=38,$$

$$M_{2}(H)=\sum_{uv\in E(H)}d_{u}d_{v}=3.2+3.2+2.2+3.2+3.2+2.2+3.2+3.2+2.2+3.2+2.2+3.2+3.2+3.2=76,$$

$$F(H)=\sum_{v\in V(H)}{\left(d_{u}\right)}^{3}=5.2^{3}+2.3^{3}=94,$$

$$H(H)=\sum_{uv\in E(H)}\frac{2}{d_{u}+d_{v}}=\frac{2}{3+2}+\frac{2}{3+2}+\frac{2}{2+2}+\frac{2}{3+2}+\frac{2}{3+2}+\frac{2}{2+2}+\frac{2}{3+2}+$$

$$\frac{2}{3+2}+\frac{2}{2+2}+\frac{2}{3+2}+\frac{2}{2+2}+\frac{2}{3+3}+\frac{2}{3+2}+\frac{2}{3+2}=5.93,$$

$$R(H)=\sum_{uv\in E(H)}\frac{1}{\sqrt{d_{u}d_{v}}}=\frac{1}{\sqrt{3.2}}+\frac{1}{\sqrt{3.2}}+\frac{1}{\sqrt{2.2}}+\frac{1}{\sqrt{3.2}}+\frac{1}{\sqrt{3.2}}+\frac{1}{\sqrt{2.2}}+\frac{1}{\sqrt{3.2}}+$$

$$\frac{1}{\sqrt{3.2}}+\frac{1}{\sqrt{2.2}}+\frac{1}{\sqrt{3.2}}+\frac{1}{\sqrt{2.2}}+\frac{1}{\sqrt{3.2}}+\frac{1}{\sqrt{3.2}}+\frac{1}{\sqrt{3.2}}=6.08$$

$$SO(H)=\sum_{uv\in E(H)}\sqrt{{\left(d_{u}\right)}^{2}+{\left(d_{v}\right)}^{2}}=\sqrt{3^{2}+2^{2}}+\sqrt{3^{2}+2^{2}}+\sqrt{2^{2}+2^{2}}+\sqrt{3^{2}+2^{2}}+\sqrt{3^{2}+2^{2}}+\sqrt{2^{2}+2^{2}}+$$

$$\sqrt{3^{2}+2^{2}}+\sqrt{3^{2}+2^{2}}+\sqrt{2^{2}+2^{2}}+\sqrt{3^{2}+2^{2}}+\sqrt{2^{2}+2^{2}}+\sqrt{3^{2}+2^{2}}+\sqrt{3^{2}+2^{2}}+\sqrt{3^{2}+2^{2}}=47.37.$$

 □

Theorem 6 *Let p, q be distinct prime numbers and* H=(V(H),E(H)) *be the PIS-hypergraph of* $Z_{p^{3}q}$ *. Then,*

$$\begin{array}{ccc} M_{1}(H)=23\; & M_{2}(H)=28\; & F(H)=53\\ H(H)=3.47\; & R(H)=3.68\; & SO(H)=21.43 \end{array}.$$

Proof Let us consider the ideals $u_{1}=pZ_{p^{3}q}$, $u_{2}=qZ_{p^{3}q}$, $u_{3}=p^{2}Z_{p^{3}q},u_{4}=pqZ_{p^{3}q},u_{5}=p^{3}Z_{p^{3}q},u_{6}=p^{2}qZ_{p^{3}q}$. Thus, in hypergraph H=(V(H),E(H)), the sets of vertices and hyperedges have the form such that $V(H)=\{u_{1}$, $u_{2}$, $u_{3}$, $u_{4},u_{5},u_{6}\}$ and $E(H)=\{E_{1}=\{u_{1},u_{3},u_{4}\}$, $E_{2}=\{u_{2},u_{4}\}$, $E_{3}=\{u_{2}$, $u_{6}\},E_{4}=\{u_{1},u_{6}\},E_{5}=\{u_{4},u_{5}\}\}$. Therefore, $d(u_{1})=2$, $d(u_{2})=2,d(u_{3})=1,d(u_{4})=3,d(u_{5})=1,d(u_{6})=2$ are obtained by vertex partition technique. Hence, we get

$$M_{1}(H)=\sum_{v\in V(H)}{\left(d_{u}\right)}^{2}=2.1^{2}+3.2^{2}+1.3^{2}=23,$$

$$M_{2}(H)=\sum_{uv\in E(H)}d_{u}d_{v}=2.1+2.3+1.3+2.3+2.2+2.2+3.1=28,$$

$$F(H)=\sum_{v\in V(H)}{\left(d_{u}\right)}^{3}=2.1^{3}+3.2^{3}+1.3^{3}=53,$$

$$H(H)=\sum_{uv\in E(H)}\frac{2}{d_{u}+d_{v}}=\frac{2}{2+1}+\frac{2}{2+3}+\frac{2}{1+3}+\frac{2}{2+3}+\frac{2}{2+2}+\frac{2}{2+2}+\frac{2}{3+1}=3.47$$

$$R(H)=\sum_{uv\in E(H)}\frac{1}{\sqrt{d_{u}d_{v}}}=\frac{1}{\sqrt{2.1}}+\frac{1}{\sqrt{2.3}}+\frac{1}{\sqrt{1.3}}+\frac{1}{\sqrt{2.3}}+\frac{1}{\sqrt{2.2}}+\frac{1}{\sqrt{2.2}}+\frac{1}{\sqrt{3.1}}=3.68$$

$$SO(H)=\sum_{uv\in E(H)}\sqrt{{\left(d_{u}\right)}^{2}+{\left(d_{v}\right)}^{2}}=\sqrt{2^{2}+1^{2}}+\sqrt{2^{2}+3^{2}}+\sqrt{1^{2}+3^{2}}+\sqrt{2^{2}+3^{2}}+\sqrt{2^{2}+2^{2}}+\sqrt{2^{2}+2^{2}}+$$

$$\sqrt{3^{2}+1^{2}}=21.43.$$

Hence, the sufficiency is clear. □

Theorem 7 *Let p, q, r be distinct prime numbers and* H=(V(H),E(H)) *be the PIS-hypergraph of* $Z_{p^{2}qr}$ *. Then,*

$$\begin{array}{ccc} M_{1}(H)=134\; & M_{2}(H)=509\; & F(H)=586\\ H(H)=8.68\; & R(H)=8.97\; & SO(H)=192.22 \end{array}.$$

Proof Let us consider the ideals $u_{1}=pZ_{p^{2}qr}$, $u_{2}=qZ_{p^{2}qr}$, $u_{3}=p^{2}Z_{p^{2}qr}$, $u_{4}=rZ_{p^{2}qr},u_{5}=pqZ_{p^{2}qr},u_{6}=prZ_{p^{2}qr}$, $u_{7}=p^{2}qZ_{p^{2}qr}$, $u_{8}=qrZ_{p^{2}qr}$, $u_{9}=p^{2}rZ_{p^{2}qr}$, $u_{10}=pqrZ_{p^{2}qr}$. Thus, in hypergraph H=(V(H),E(H)), there exist vertices and hyperedges as follows which are obtained by vertex and edge partition technique.

**Table:** 

Hyperedge List			
*E*_1_ = {*u*_1_,*u*_3_,*u*_5_,*u*_6_}	*E*_2_ = {*u*_2_,*u*_5_,*u*_8_}	*E*_3_ = {*u*_3_,*u*_10_}	*E*_4_ = {*u*_4_,*u*_6_,*u*_8_}
*E*_5_ = {*u*_5_,*u*_6_,*u*_8_}	*E*_6_ = {*u*_5_,*u*_8_,*u*_9_}	*E*_7_ = {*u*_6_,*u*_8_,*u*_9_	*E*_8_ = {*u*_1_,*u*_5_,*u*_9_}
*E*_9_ = {*u*_4_,*u*_8_,*u*_9_}	*E*_10_ = {*u*_1_,*u*_3_,*u*_10_}	*E*_11_ = {*u*_2_,*u*_10_}	*E*_12_ = {*u*_4_,*u*_10_}

and

**Table:** 

Degree List of the Vertices				
*d*(*u*_1_)=1	*d*(*u*_2_)=2	*d*(*u*_3_)=3	*d*(*u*_4_)=3	*d*(*u*_5_)=5
*d*(*u*_6_)=4	*d*(*u*_7_)=1	*d*(*u*_8_)=6	*d*(*u*_9_)=3	*d*(*u*_10_)=4

Then,

$$M_{1}(H)=\sum_{v\in V(H)}{\left(d_{u}\right)}^{2}=1.1^{2}+1.2^{2}+4.3^{2}+2.4^{2}+1.5^{2}+1.6^{2}=134,$$

$$M_{2}(H)=\sum_{uv\in E(H)}d_{u}d_{v}=3.3+3.5+3.4+3.5+3.4+5.4+2.5+2.6+5.6+3.4+3.4+3.6+4.6+5.4+5.6+$$

4.6+5.6+5.3+6.3+4.1+4.6+1.6+3.5+3.3+5.3+3.6+3.3+3.3+6.3+3.4+3.4+2.4+3.4=509,

$$F(H)=\sum_{v\in V(H)}{\left(d_{u}\right)}^{3}=1.1^{3}+1.2^{3}+4.3^{3}+2.4^{3}+1.5^{3}+1.6^{3}=586,$$

$$H(H)=\sum_{uv\in E(H)}\frac{2}{d_{u}+d_{v}}=\frac{2}{3+3}+\frac{2}{3+5}+\frac{2}{3+4}+\frac{2}{3+5}+\frac{2}{3+4}+\frac{2}{5+4}+\frac{2}{2+5}+\frac{2}{2+6}+\frac{2}{5+6}+\frac{2}{3+4}+$$

$$\frac{2}{3+4}+\frac{2}{3+6}+\frac{2}{4+6}+\frac{2}{5+4}+\frac{2}{5+6}+\frac{2}{4+6}+\frac{2}{5+6}+\frac{2}{5+3}+\frac{2}{6+3}+\frac{2}{4+1}+\frac{2}{4+6}+\frac{2}{1+6}+\frac{2}{3+5}+$$

$$\frac{2}{3+3}+\frac{2}{5+3}+\frac{2}{3+6}+\frac{2}{3+3}+\frac{2}{3+3}+\frac{2}{6+3}+\frac{2}{3+4}+\frac{2}{3+4}+\frac{2}{2+4}+\frac{2}{3+4}=8.68$$

$$R(H)=\sum_{uv\in E(H)}\frac{1}{\sqrt{d_{u}d_{v}}}=\frac{1}{\sqrt{3.3}}+\frac{1}{\sqrt{3.5}}+\frac{1}{\sqrt{3.4}}+\frac{1}{\sqrt{3.5}}+\frac{1}{\sqrt{3.4}}+\frac{1}{\sqrt{5.4}}+\frac{1}{\sqrt{2.5}}+\frac{1}{\sqrt{2.6}}+\frac{1}{\sqrt{5.6}}+\frac{1}{\sqrt{3.4}}+$$

$$\frac{1}{\sqrt{3.4}}+\frac{1}{\sqrt{3.6}}+\frac{1}{\sqrt{4.6}}+\frac{1}{\sqrt{5.4}}+\frac{1}{\sqrt{5.6}}+\frac{1}{\sqrt{4.6}}+\frac{1}{\sqrt{5.6}}+\frac{1}{\sqrt{5.3}}+\frac{1}{\sqrt{6.3}}+\frac{1}{\sqrt{4.1}}+\frac{1}{\sqrt{4.6}}+\frac{1}{\sqrt{1.6}}+\frac{1}{\sqrt{3.5}}+$$

$$\frac{1}{\sqrt{3.3}}+\frac{1}{\sqrt{5.3}}+\frac{1}{\sqrt{3.6}}+\frac{1}{\sqrt{3.3}}+\frac{1}{\sqrt{3.3}}+\frac{1}{\sqrt{6.3}}+\frac{1}{\sqrt{3.4}}+\frac{1}{\sqrt{3.4}}+\frac{1}{\sqrt{2.4}}+\frac{1}{\sqrt{3.4}}=8.97$$

$$SO(H)=\sum_{uv\in E(H)}\sqrt{{\left(d_{u}\right)}^{2}+{\left(d_{v}\right)}^{2}}=\sqrt{3^{2}+3^{2}}+\sqrt{3^{2}+5^{2}}+\sqrt{3^{2}+4^{2}}+\sqrt{3^{2}+5^{2}}+\sqrt{3^{2}+4^{2}}+\sqrt{5^{2}+4^{2}}+$$

$$\sqrt{2^{2}+5^{2}}+\sqrt{2^{2}+6^{2}}+\sqrt{5^{2}+6^{2}}+\sqrt{3^{2}+4^{2}}+\sqrt{3^{2}+4^{2}}+\sqrt{3^{2}+6^{2}}+\sqrt{4^{2}+6^{2}}+\sqrt{5^{2}+4^{2}}+\sqrt{5^{2}+6^{2}}+$$

$$\sqrt{4^{2}+6^{2}}+\sqrt{5^{2}+6^{2}}+\sqrt{5^{2}+3^{2}}+\sqrt{6^{2}+3^{2}}+\sqrt{4^{2}+1^{2}}+\sqrt{4^{2}+6^{2}}+\sqrt{1^{2}+6^{2}}+\sqrt{3^{2}+5^{2}}+\sqrt{3^{2}+3^{2}}+$$

$$\sqrt{5^{2}+3^{2}}+\sqrt{3^{2}+6^{2}}+\sqrt{3^{2}+3^{2}}+\sqrt{3^{2}+3^{2}}+\sqrt{6^{2}+3^{2}}+\sqrt{3^{2}+4^{2}}+\sqrt{3^{2}+4^{2}}+\sqrt{2^{2}+4^{2}}+\sqrt{3^{2}+4^{2}}$$

=192.22

Hence, the sufficiency is clear. □

Theorem 8 *Let p, q, r, s be distinct prime numbers and* H=(V(H),E(H)) *be the PIS-hypergraph of* $Z_{pqrs}$ *. Then,*

$$\begin{array}{ccc} M_{1}(H)=761\; & M_{2}(H)=6799\; & F(H)=7001\\ H(H)=14.65\; & R(H)=15.27\; & SO(H)=1260.92 \end{array}.$$

Proof Let us consider the non-trivial ideals $u_{1}=pZ_{pqrs}$, $u_{2}=qZ_{pqrs}$, $u_{3}=rZ_{pqrs}$, $u_{4}=pqZ_{pqrs},u_{5}=sZ_{pqrs},u_{6}=prZ_{pqrs}$, $u_{7}=psZ_{pqrs}$, $u_{8}=qrZ_{pqrs}$, $u_{9}=qsZ_{pqrs}$, $u_{10}=pqrZ_{pqrs}$, $u_{11}=rsZ_{pqrs}$, $u_{12}=pqsZ_{pqrs}$, $u_{13}=prsZ_{pqrs}$, $u_{14}=qrsZ_{pqrs}$ where $u_{1}$, $u_{2}$, $u_{3}$, $u_{5}$ are prime ones at that time. Thus in hypergraph H=(V(H),E(H)) taking into account the set of vertices $V(H)=\{u_{1}$, $u_{2}$, $u_{3}$, $u_{4},u_{5},u_{6},u_{7},u_{8},u_{9},u_{10},u_{11},u_{12},u_{13},u_{14}\}$, degrees of the vertices and partition of the hyperedges are obtained as follows:

**Table:** 

Hyperedge List				
*E*_1_ = {*u*_1_,*u*_4_,*u*_6_,*u*_7_}	*E*_2_ = {*u*_1_,*u*_4_,*u*_13_}	*E*_3_ = {*u*_1_, *u*_6_,*u*_12_}	*E*_4_ = {*u*_1_,*u*_7_,*u*_10_}	*E*_5_ = {*u*_2_,*u*_4_,*u*_8_,*u*_9_}
*E*_6_ = {*u*_2_,*u*_4_,*u*_14_}	*E*_7_ = {*u*_2_,*u*_8_,*u*_12_}	*E*_8_ = {*u*_2_,*u*_9_,*u*_10_}	*E*_9_ = {*u*_3_,*u*_6_,*u*_8_,*u*_11_}	*E*_10_ = {*u*_3_,*u*_8_,*u*_13_}
*E*_11_ = {*u*_3_,*u*_10_,*u*_11_}	*E*_12_ = {*u*_3_,*u*_6_,*u*_14_}	*E*_13_ = {*u*_4_,*u*_6_,*u*_7_,*u*_14_}	*E*_14_ = {*u*_4_,*u*_7_,*u*_9_}	*E*_15_ = {*u*_4_,*u*_8_,*u*_9_,*u*_13_}
*E*_16_ = {*u*_5_,*u*_7_,*u*_9_}	*E*_17_ = {*u*_5_,*u*_7_,*u*_11_}	*E*_18_ = {*u*_5_,*u*_9_,*u*_11_}	*E*_19_ = {*u*_5_,*u*_9_,*u*_13_}	*E*_20_ = {*u*_5_,*u*_11_,*u*_12_}
*E*_21_ = {*u*_6_,*u*_7_,*u*_14_}	*E*_22_ = {*u*_6_,*u*_8_,*u*_11_,*u*_12_}	*E*_23_ = {*u*_6_,*u*_7_,*u*_11_}	*E*_24_ = {*u*_7_,*u*_9_,*u*_10_}	*E*_25_ = {*u*_5_,*u*_7_,*u*_14_}
*E*_26_ = {*u*_8_,*u*_9_,*u*_11_}	*E*_27_ = {*u*_8_,*u*_9_,*u*_13_}	*E*_28_ = {*u*_9_,*u*_10_,*u*_11_}	*E*_29_ = {*u*_7_,*u*_9_,*u*_11_}	*E*_30_ = {*u*_7_,*u*_10_,*u*_11_}

and

**Table:** 

Degree List of the Vertices						
*d*(*u*_1_)=4	*d*(*u*_2_)=4	*d*(*u*_3_)=4	*d*(*u*_4_)=7	*d*(*u*_5_)=6	*d*(*u*_6_)=8	*d*(*u*_7_)=12
*d*(*u*_8_)=8	*d*(*u*_9_)=12	*d*(*u*_10_)=5	*d*(*u*_11_)=11	*d*(*u*_12_)=4	*d*(*u*_13_)=5	*d*(*u*_14_)=5

$$M_{1}(H)=\sum_{v\in V(H)}{\left(d_{u}\right)}^{2}=4.4^{2}+3.5^{2}+1.6^{2}+1.7^{2}+2.8^{2}+1.11^{2}+2.12^{2}=761,$$

$$M_{2}(H)=\sum_{uv\in E(H)}d_{u}d_{v}=4.7+4.8+4.12+7.8+7.12+8.12+4.5+4.7+7.5+4.8+4.4+8.4+4.12+4.5+$$

12.5+4.7+4.8+4.12+7.8+7.12+8.12+4.7+4.5+7.5+4.8+4.4+8.4+4.12+4.5+12.5+4.8+4.8

+4.11+8.8+8.11+8.11+4.8+4.5+8.5+4.5+4.11+5.11+4.8+4.5+8.5+4.5+8.5+7.8+8.5+12.5

+8.12+7.12+7.5+7.12+7.12+12.12+7.8+7.12+7.5+8.12+8.5+12.5+6.12+6.12+12.12+6.12

+6.11+12.11+6.12+6.11+12.11+6.12+12.5+6.5+6.11+6.4+11.4+8.12+12.5+8.5+8.8+8.11

+8.4+8.11+8.4+11.4+8.12+8.11+12.11+12.12+12.5+12.5+6.12+6.5+12.5+8.12+8.11+12.11

+8.12+8.5+12.5+12.5+12.11+5.11+12.12+12.11+12.11+12.5+12.11+5.11=6799,

$$F(H)=\sum_{v\in V(H)}{\left(d_{u}\right)}^{2}=4.4^{3}+3.5^{3}+1.6^{3}+1.7^{3}+2.8^{3}+1.11^{3}+2.12^{3}=7001,$$

$$H(H)=\sum_{uv\in E(H)}\frac{2}{d_{u}+d_{v}}=\frac{2}{4+7}+\frac{2}{4+8}+\frac{2}{4+12}+\frac{2}{7+8}+\frac{2}{7+12}+\frac{2}{8+12}+\frac{2}{4+7}+\frac{2}{4+5}+\frac{2}{7+5}+\frac{2}{4+8}$$

$$+\frac{2}{4+4}+\frac{2}{8+4}+\frac{2}{4+4}+\frac{2}{8+4}+\frac{2}{4+12}+\frac{2}{4+5}+\frac{2}{12+5}+\frac{2}{4+7}+\frac{2}{4+8}+\frac{2}{4+12}+\frac{2}{7+8}+\frac{2}{7+12}+\frac{2}{8+12}$$

$$+\frac{2}{4+7}+\frac{2}{4+5}+\frac{2}{7+5}+\frac{2}{4+8}+\frac{2}{4+4}+\frac{2}{8+4}+\frac{2}{4+12}+\frac{2}{4+5}+\frac{2}{12+5}+\frac{2}{4+8}+\frac{2}{4+8}+\frac{2}{4+11}+\frac{2}{8+8}$$

$$+\frac{2}{8+11}+\frac{2}{8+11}+\frac{2}{4+8}+\frac{2}{4+5}+\frac{2}{7+12}+\frac{2}{8+5}+\frac{2}{4+5}+\frac{2}{4+11}+\frac{2}{5+11}+\frac{2}{4+8}+\frac{2}{4+5}+\frac{2}{8+5}+\frac{2}{7+8}$$

$$+\frac{2}{7+12}+\frac{2}{7+5}+\frac{2}{8+12}+\frac{2}{8+5}+\frac{2}{7+12}+\frac{2}{7+12}+\frac{2}{12+12}+\frac{2}{7+8}+\frac{2}{7+5}+\frac{2}{8+12}+\frac{2}{8+5}+\frac{2}{12+5}$$

$$+\frac{2}{6+12}+\frac{2}{6+12}+\frac{2}{12+12}+\frac{2}{6+12}+\frac{2}{6+11}+\frac{2}{12+11}+\frac{2}{6+12}+\frac{2}{6+11}+\frac{2}{12+5}+\frac{2}{12+11}+\frac{2}{6+12}+\frac{2}{6+5}$$

$$+\frac{2}{12+5}+\frac{2}{6+11}+\frac{2}{6+4}+\frac{2}{11+4}+\frac{2}{8+12}+\frac{2}{8+5}+\frac{2}{12+5}+\frac{2}{8+8}+\frac{2}{8+11}+\frac{2}{8+4}+\frac{2}{8+11}+\frac{2}{8+4}$$

$$+\frac{2}{11+4}+\frac{2}{8+12}+\frac{2}{8+11}+\frac{2}{12+11}+\frac{2}{12+12}+\frac{2}{12+5}+\frac{2}{12+5}+\frac{2}{6+12}+\frac{2}{6+5}+\frac{2}{12+5}+\frac{2}{8+12}$$

$$+\frac{2}{8+11}+\frac{2}{12+11}+\frac{2}{8+12}+\frac{2}{8+5}+\frac{2}{12+5}+\frac{2}{12+5}+\frac{2}{12+11}+\frac{2}{5+11}+\frac{2}{12+12}+\frac{2}{12+11}+\frac{2}{12+11}$$

$$+\frac{2}{12+5}+\frac{2}{12+11}+\frac{2}{5+11}=14.65,$$

$$R(H)=\sum_{uv\in E(H)}\frac{1}{\sqrt{d_{u}d_{v}}}=\frac{1}{\sqrt{4.7}}+\frac{1}{\sqrt{4.8}}+\frac{1}{\sqrt{4.12}}+\frac{1}{\sqrt{7.8}}+\frac{1}{\sqrt{7.12}}+\frac{1}{\sqrt{8.12}}+\frac{1}{\sqrt{4.7}}+\frac{1}{\sqrt{4.5}}+\frac{1}{\sqrt{7.5}}+\frac{1}{\sqrt{4.8}}+$$

$$\frac{1}{\sqrt{4.4}}+\frac{1}{\sqrt{8.4}}+\frac{1}{\sqrt{4.4}}+\frac{1}{\sqrt{8.4}}+\frac{1}{\sqrt{4.12}}+\frac{1}{\sqrt{4.5}}+\frac{1}{\sqrt{12.5}}+\frac{1}{\sqrt{4.7}}+\frac{1}{\sqrt{4.8}}+\frac{1}{\sqrt{4.12}}+\frac{1}{\sqrt{7.8}}+\frac{1}{\sqrt{7.12}}+\frac{1}{\sqrt{8.12}}+$$

$$\frac{1}{\sqrt{4.7}}+\frac{1}{\sqrt{4.5}}+\frac{1}{\sqrt{7.5}}+\frac{1}{\sqrt{4.8}}+\frac{1}{\sqrt{4.4}}+\frac{1}{\sqrt{8.4}}+\frac{1}{\sqrt{4.12}}+\frac{1}{\sqrt{4.5}}+\frac{1}{\sqrt{12.5}}+\frac{1}{\sqrt{4.8}}+\frac{1}{\sqrt{4.8}}+\frac{1}{\sqrt{4.11}}+\frac{1}{\sqrt{8.8}}+$$

$$\frac{1}{\sqrt{8.11}}+\frac{1}{\sqrt{8.11}}\frac{1}{\sqrt{4.8}}+\frac{1}{\sqrt{4.5}}+\frac{1}{\sqrt{7.12}}+\frac{1}{\sqrt{8.5}}+\frac{1}{\sqrt{4.5}}+\frac{1}{\sqrt{4.11}}+\frac{1}{\sqrt{5.11}}+\frac{1}{\sqrt{4.8}}+\frac{1}{\sqrt{4.5}}+\frac{1}{\sqrt{8.5}}+\frac{1}{\sqrt{7.8}}+$$

$$\frac{1}{\sqrt{7.12}}+\frac{1}{\sqrt{7.5}}+\frac{1}{\sqrt{8.12}}+\frac{1}{\sqrt{8.5}}+\frac{1}{\sqrt{7.12}}+\frac{1}{\sqrt{7.12}}+\frac{1}{\sqrt{12.12}}+\frac{1}{\sqrt{7.8}}+\frac{1}{\sqrt{7.5}}+\frac{1}{\sqrt{8.12}}+\frac{1}{\sqrt{8.5}}+\frac{1}{\sqrt{12.5}}+$$

$$\frac{1}{\sqrt{6.12}}+\frac{1}{\sqrt{6.12}}+\frac{1}{\sqrt{12.12}}+\frac{1}{\sqrt{6.12}}+\frac{1}{\sqrt{6.11}}+\frac{1}{\sqrt{12.11}}+\frac{1}{\sqrt{6.12}}+\frac{1}{\sqrt{6.11}}+\frac{1}{\sqrt{12.5}}+\frac{1}{\sqrt{12.11}}+\frac{1}{\sqrt{6.12}}+$$

$$\frac{1}{\sqrt{6.5}}+\frac{1}{\sqrt{12.5}}+\frac{1}{\sqrt{6.11}}+\frac{1}{\sqrt{6.4}}+\frac{1}{\sqrt{11.4}}+\frac{1}{\sqrt{8.12}}+\frac{1}{\sqrt{8.5}}+\frac{1}{\sqrt{12.5}}+\frac{1}{\sqrt{8.8}}+\frac{1}{\sqrt{8.11}}+\frac{1}{\sqrt{8.4}}+\frac{1}{\sqrt{8.11}}+$$

$$\frac{1}{\sqrt{8.4}}+\frac{1}{\sqrt{11.4}}+\frac{1}{\sqrt{8.12}}+\frac{1}{\sqrt{8.11}}+\frac{1}{\sqrt{12.11}}+\frac{1}{\sqrt{12.12}}+\frac{1}{\sqrt{12.5}}+\frac{1}{\sqrt{12.5}}+\frac{1}{\sqrt{6.12}}+\frac{1}{\sqrt{6.5}}+\frac{1}{\sqrt{12.5}}+\frac{1}{\sqrt{8.12}}+$$

$$\frac{1}{\sqrt{8.11}}+\frac{1}{\sqrt{12.11}}\frac{1}{\sqrt{8.11}}+\frac{1}{\sqrt{12.11}}+\frac{1}{\sqrt{5.11}}=15.27,$$

$$SO(H)=\sum_{uv\in E(H)}\sqrt{{\left(d_{u}\right)}^{2}+{\left(d_{v}\right)}^{2}}=\sqrt{4^{2}+7^{2}}+\sqrt{4^{2}+8^{2}}+\sqrt{4^{2}+12^{2}}+\sqrt{7^{2}+8^{2}}+\sqrt{7^{2}+12^{2}}+\sqrt{8^{2}+12^{2}}+$$

$$\sqrt{4^{2}+7^{2}}+\sqrt{4^{2}+5^{2}}+\sqrt{7^{2}+5^{2}}+\sqrt{4^{2}+8^{2}}+\sqrt{4^{2}+4^{2}}+\sqrt{8^{2}+4^{2}}+\sqrt{4^{2}+12^{2}}+\sqrt{4^{2}+5^{2}}+\sqrt{12^{2}+5^{2}}+$$

$$\sqrt{4^{2}+7^{2}}+\sqrt{4^{2}+8^{2}}+\sqrt{4^{2}+12^{2}}+\sqrt{7^{2}+8^{2}}+\sqrt{7^{2}+12^{2}}+\sqrt{8^{2}+12^{2}}+\sqrt{4^{2}+7^{2}}+\sqrt{4^{2}+5^{2}}+\sqrt{7^{2}+5^{2}}+$$

$$\sqrt{4^{2}+8^{2}}+\sqrt{4^{2}+4^{2}}+\sqrt{8^{2}+4^{2}}+\sqrt{4^{2}+12^{2}}+\sqrt{4^{2}+5^{2}}+\sqrt{12^{2}+5^{2}}+\sqrt{4^{2}+8^{2}}+\sqrt{4^{2}+8^{2}}+\sqrt{4^{2}+11^{2}}+$$

$$\sqrt{8^{2}+8^{2}}+\sqrt{8^{2}+11^{2}}+\sqrt{8^{2}+11^{2}}+\sqrt{4^{2}+8^{2}}+\sqrt{4^{2}+5^{2}}+\sqrt{8^{2}+5^{2}}+\sqrt{4^{2}+5^{2}}+\sqrt{4^{2}+11^{2}}+\sqrt{5^{2}+11^{2}}+$$

$$\sqrt{4^{2}+8^{2}}+\sqrt{4^{2}+5^{2}}+\sqrt{8^{2}+5^{2}}+\sqrt{7^{2}+8^{2}}+\sqrt{7^{2}+12^{2}}+\sqrt{7^{2}+5^{2}}+\sqrt{8^{2}+12^{2}}+\sqrt{8^{2}+5^{2}}+$$

$$\sqrt{12^{2}+5^{2}}+\sqrt{7^{2}+12^{2}}+\sqrt{7^{2}+12^{2}}+\sqrt{12^{2}+12^{2}}+\sqrt{7^{2}+8^{2}}+\sqrt{7^{2}+12^{2}}+\sqrt{7^{2}+5^{2}}+\sqrt{8^{2}+12^{2}}+$$

$$\sqrt{8^{2}+5^{2}}+\sqrt{12^{2}+5^{2}}+\sqrt{6^{2}+12^{2}}+\sqrt{6^{2}+12^{2}}+\sqrt{12^{2}+12^{2}}+\sqrt{6^{2}+12^{2}}+\sqrt{6^{2}+11^{2}}+\sqrt{12^{2}+11^{2}}+$$

$$\sqrt{6^{2}+12^{2}}+\sqrt{6^{2}+11^{2}}+\sqrt{12^{2}+11^{2}}+\sqrt{6^{2}+12^{2}}+\sqrt{6^{2}+5^{2}}+\sqrt{12^{2}+5^{2}}+\sqrt{6^{2}+11^{2}}+\sqrt{6^{2}+4^{2}}+$$

$$\sqrt{11^{2}+4^{2}}+\sqrt{8^{2}+12^{2}}+\sqrt{8^{2}+5^{2}}+\sqrt{12^{2}+5^{2}}+\sqrt{12^{2}+5^{2}}+\sqrt{8^{2}+8^{2}}+\sqrt{8^{2}+11^{2}}+\sqrt{8^{2}+4^{2}}+$$

$$\sqrt{8^{2}+11^{2}}+\sqrt{8^{2}+4^{2}}+\sqrt{11^{2}+4^{2}}+\sqrt{8^{2}+12^{2}}+\sqrt{8^{2}+11^{2}}+\sqrt{12^{2}+11^{2}}+\sqrt{12^{2}+12^{2}}+\sqrt{12^{2}+5^{2}}+$$

$$\sqrt{12^{2}+5^{2}}+\sqrt{6^{2}+12^{2}}+\sqrt{6^{2}+5^{2}}+\sqrt{12^{2}+5^{2}}+\sqrt{8^{2}+12^{2}}+\sqrt{8^{2}+11^{2}}+\sqrt{12^{2}+11^{2}}+\sqrt{8^{2}+12^{2}}+$$

$$\sqrt{8^{2}+5^{2}}+\sqrt{12^{2}+5^{2}}+\sqrt{12^{2}+5^{2}}+\sqrt{12^{2}+11^{2}}+\sqrt{5^{2}+11^{2}}+\sqrt{12^{2}+12^{2}}+\sqrt{12^{2}+11^{2}}+\sqrt{12^{2}+11^{2}}+$$

$$\sqrt{12^{2}+5^{2}}+\sqrt{12^{2}+11^{2}}+\sqrt{5^{2}+11^{2}}=1260.92.$$

 □

## 4 Conclusion

**Figure 4:**
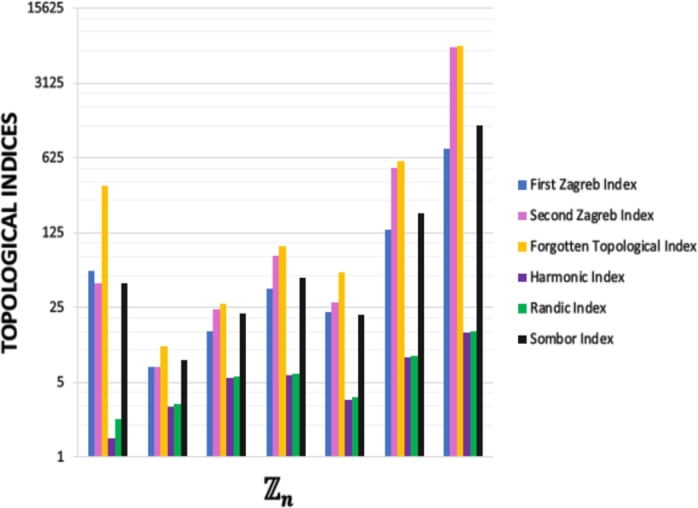
Topological Indices of *PISH* Structure.

**Figure 5:**
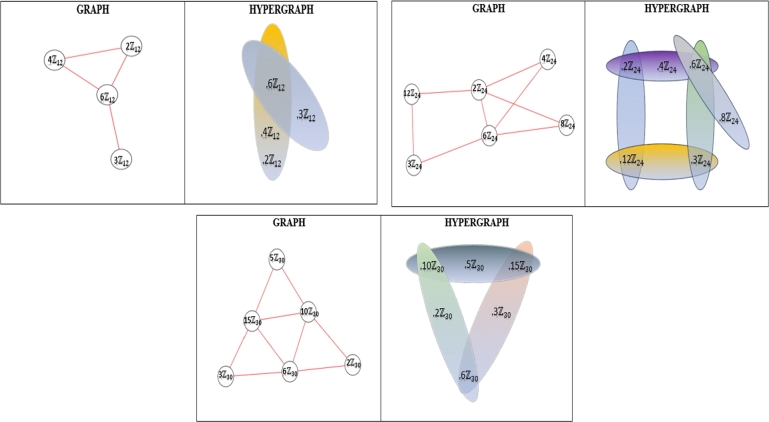
Comparison of *PIS* Graph and Hypergraph Representations.

In this study, we introduced and studied a general version of prime ideal sum graphs. We computed some degree-based topological indices for this new defined algebraic hypergraph based on prime ideal sum and computed them for $Z_{n}$ where $n=p^{\alpha },pq,p^{2}q,p^{2}q^{2},pqr,p^{3}q$, $p^{2}qr,pqrs$ for the distinct primes p,q,r and *s*. In Fig. 4, it can be seen that forgotten topological index is the maximum one and harmonic topological index is the minimum one between the other index values. Also $H(H)<R(H)<M_{1}(H)<M_{2}<F(H)$ is obtained apart from $n=p^{\alpha },p^{2}q$. Moreover, we present three specicifik graph/hypergraph representation for prime ideal sum graph/hypergraph of $Z_{n}$ (n=12,24,30; respectively) in Fig. 5. In the light of comparisonal data getting from this study, these newly defined algebraic hypergraph can be used and developed by researchers in future, for example in the social network analysis and in the other applied sciences such as cryptology, code theory and mechanics as it is mentioned above. Until very recently, algebraic concepts such as groups, rings and modules were known as abstract concepts that could be defined and whose theoretical properties were shown, but thanks to algebraic graph theory, it has been realized that visible modeling of algebraic structures can be made according to the graph model taken as a basis. However, it is made possible in our article that this modeling can be further generalized with hypergraph representation. And even the calculation of topological parameters has shed light on the topology of algebraic structures. As a result, algebraic structures that were thought to be trapped within the impenetrable walls of theory have shown that they can also take place in the applied field.

## Funding

This study was carried out with financial support from 10.13039/501100004242Princess Nourah Bint Abdulrahman University (PNU), Riyadh, Saudi Arabia.

## CRediT authorship contribution statement

**Amal S. Alali:** Writing – review & editing, Funding acquisition. **Esra Öztürk Sözen:** Writing – original draft. **Cihat Abdioğlu:** Supervision. **Shakir Ali:** Conceptualization. **Elif Eryaşar:** Visualization.

## Declaration of Competing Interest

The authors declare that they have no known competing financial interests or personal relationships that could have appeared to influence the work reported in this paper.

## Data Availability

No data were used to support the findings of this study.
